# Psychometric Properties of the Multidimensional Temperance Scale in Adolescents

**DOI:** 10.3390/ijerph182312727

**Published:** 2021-12-02

**Authors:** Fernanda Inéz García-Vázquez, Angel Alberto Valdés-Cuervo, Alma Georgina Navarro-Villarreal, Lizeth Guadalupe Parra-Pérez, Maria Fernanda Durón-Ramos, Daniela Fimbres-Celaya

**Affiliations:** 1 Department of Education, Technological Institute of Sonora, Obregon 85000, Mexico; angel.valdes@itson.edu.mx (A.A.V.-C.); gina.itson@gmail.com (A.G.N.-V.); lizpara@gmail.com (L.G.P.-P.); maria.duron@itson.edu.mx (M.F.D.-R.); 2 Department of Social Sciences, University of Sonora, Hermosillo 83000, Mexico; dafimbres96@gmail.com

**Keywords:** temperance, virtues, character strengths, positive psychology, validity, adolescence

## Abstract

Recent research has shown the relevance of measuring the virtue of temperance. The present study tested a multidimensional and second-order structure scale to assess temperance using a sub-scale of the Values in Action Inventory of Strengths for Youth (VIA-Youth). Scale properties were tested using data from a sample of 860 adolescents aged from 12 to 18 years old (*M* = 14.28 years, *SD* = 1.65). The sample was randomly split into two subsamples for model cross-validation. Using the first sample, we assessed scale dimensionality, measurement invariance, and discriminant and concurrent validity. A second sample was used for model cross-validation. Confirmatory factorial analysis confirmed the fit of one second-order factor temperance virtue model, with the dimensions of forgiveness, modesty, prudence, and self-control. The results indicate scale measurement equivalence across gender and stage of adolescence (early vs. middle). Latent means difference tests showed significant differences in forgiveness, modesty, and self-regulation by gender, and modesty according to adolescence stage. Moreover, the scale showed discriminant and concurrent validity. These findings indicate that this scale is helpful for assessing temperance in adolescents and suggest the value of temperance as a multidimensional and second-order construct.

## 1. Introduction

Virtues are central attributes that are highly appreciated in philosophy and religious theories worldwide, since they favor the optimal functioning of people [1,2]. Temperance, one of these identified virtues [3,4], contributes to a wide variety of positive consequences, such as individuals’ well-being and the achievement of goals [5,6,7]. As a result, the interest among scholars in measuring this virtue has seen exponential growth in recent years [8,9,10,11].

Temperance involves regulating emotions, behavior, and motivation [2,12]. According to the literature [2,13], this virtue encompasses the strengths of modesty (avoiding flaunting and permitting personal accomplishments to speak for themselves), self-regulation (regulating behaviors and feelings), forgiveness (leaving aside anger or revenge towards the offender), and prudence (being cautious with individual decisions and avoiding actions one may regret). Some scholars have adopted the positive psychology approach [6,14] to research this virtue because the approach embraces the scientific study of positive human functioning and adaptive behaviors at all levels, such as personal, relational, and institutional [1,15].

### 1.1. Measures of Temperance

Temperance is recognized as a crucial trait related to adolescents’ personal and academic positive outcomes [16,17,18,19]. The growing interest in studying virtues in adolescence led to the development of the Values in Action Inventory of Strengths for Youth (VIA-Youth) [20]. This measure, which has been widely used, includes a subscale of the virtue of temperance [21,22,23]; the subscale consists of four first-order factor measures that include forgiveness, modesty, prudence, and self-regulation [20]. However, research shows that the factorial structure of the scale is inconsistent. That is, some studies reported it as a three-factor scale [9,23] and others [20,21] reported it as a four-factor, five-factor [24], or even six-factor scale [19]. In addition, a study conducted by Van Eeden et al. [22] showed no clustering of the strengths, contradicting the theory. Second, the evidence for second-order models is scarce [21,23]. Furthermore, most studies have conducted exploratory factor analyses or principal component analysis [9,19,24], using total strength scores instead of the items of the scale. As a result, the factor weights of each item were not reported. Finally, studies conducted within the Mexican context are limited, and have only focused on the adult population [25,26].

### 1.2. Measurement Invariance

Although the empirical evidence is still inconclusive, the current literature suggests that temperance differs by gender and age. Some studies [21,27,28,29,30] report higher scores of temperance in males, whereas others [19,31,32,33] report higher levels in females. Similarly, findings regarding age are contradictory, whereas some studies indicate that temperance positively correlates with age [10,31,32,34] and others have found no association between these variables [35,36] and it has been recently found that temperance decreases in adolescence. However, these findings should be taken with caution since these studies did not report measurement invariance when examining group differences. Verifying measurement invariance results is necessary to make a meaningful comparison between group means and to warrant that group differences are associated with latent variables [37,38]. Therefore, it is essential to examine the measurement equivalence of the temperance scale by gender and stage of adolescence to realize meaningful comparisons by groups in temperance dimensions of self-control, forgiveness, prudence, and modesty.

### 1.3. Concurrent Validity

Proactive aggression is planned, not provoked, and directed toward obtaining a goal or benefit [39,40,41]. Reactive aggression is anger that is motivated and an unregulated response to perceived threats [42,43,44]. Studies suggest that the strengths of temperance hinder aggression [45,46]. For example, findings have indicated that modesty [47,48,49,50], self-regulation [51,52,53,54], forgiveness [45,55,56,57,58,59], and prudence [60] are associated with both proactive and reactive bullying.

### 1.4. The Present Study

**Figure 1:**
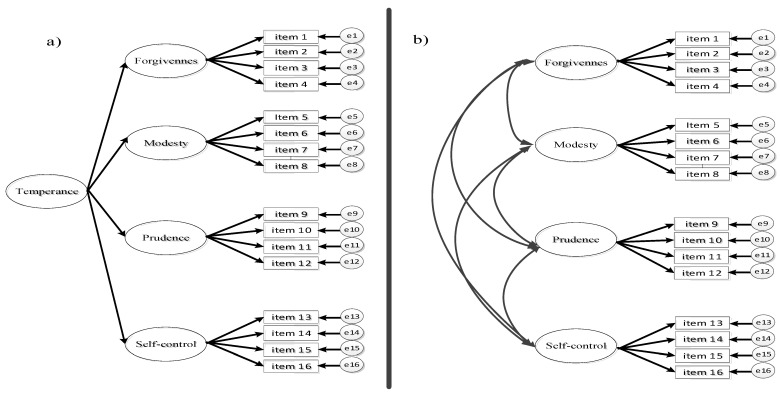
Factor model of temperance depicting one second-order factor structure hypothesized to underlie the four first-order factors; e—error; item—question of the temperance scale; (**a**)—second-order model; (**b**)—four-first-order factors model.

The measurement of temperance has some potential weaknesses, such as (a) the dearth of studies that have examined the fit to the data of a second-order factor model; (b) no study known by the authors has examined the invariance of measurement according to gender and stage of adolescence, although prior research suggests that temperance may differ by gender and age [27,32]; (c) the studies evaluating the discriminant and concurrent validity of temperance are scarce; (d) there is no study known by the authors that has examined the psychometric properties of a multidimensional temperance scale in Mexican adolescents. To attend to these gaps, in this study we proposed: (1) examining the dimensionality of a second-order model that displays four first-order factors (see Figure 1; see Table 1); (2) examining scale measurement invariance by gender and adolescence stage (early vs. middle); (3) comparing latent variable mean differences across groups, if scale measurement invariance is confirmed; (4) assessing discriminant validity by analyzing the relationships between each subscale; and (5) examining concurrent validity by testing the correlations between the dimensions of the temperance scale and bullying aggression (proactive and reactive).

To accomplish these purposes, we considered five hypotheses. Hypothesis 1 (internal structure): the indicators used to measure temperance reveal a second-order factor structure that contains four first-order factors (forgiveness, modesty, prudence, and self-control) that fit the data. Hypothesis 2 (measurement invariance): the scale shows robust invariance across gender and adolescence stages. Hypothesis 3 (latent means): Studies are not conclusive, and no previous hypothesis about gender and stage of adolescence differences was considered. Hypothesis 4 (discriminant validity): Each subscale of the temperance scale discriminates between conceptually similar constructs. Hypothesis 5 (concurrent validity): the dimensions of the temperance scale have a negative relation with proactive and reactive bullying aggression.

## 2. Materials and Methods

### 2.1. Participants

Participants were students from 32 public secondary and 32 high schools from three cities in Sonora, Mexico. These schools typically serve students of low and middle socioeconomic status. The study sample was composed of 860 adolescent students, 406 (47.2%) males and 454 (52.8%) females, whose ages ranged from 12 to 18 years old; 430 (50%) early adolescents (*M* age = 12.79 years, *SD* = 0.07) and 430 (50%) middle adolescents (*M* age = 16.58 years, *SD* = 0.06). The sample was randomly split into two subsamples for model calibration (*n* = 430) and cross-validation (*n* = 430).

### 2.2. Measures

#### 2.2.1. Temperance

A subscale of temperance virtue (TV) of the Values in Action Inventory of Strengths for Youth [20] (VIA-Youth; Spanish version) was used; temperance is a virtue that encompasses strengths that focus on controlling excesses. The scale includes four dimensions: forgiveness, which involves leaving aside resentment or revenge and a benevolent feeling towards the offender (4 items, e.g., I am a forgiving person); modesty, which implies avoiding flaunting and permitting personal accomplishments to provide the necessary information about oneself (4 items, e.g., I never brag or flaunt my accomplishments); prudence, which includes being careful with personal decisions and avoiding speaking or behaving in a way that may be regretted (4 items, e.g., I think about the consequences of my behavior before I act.); and self-regulation, which involves the ability to regulate actions, emotions and resist temptations (4 items, e.g., I can control my anger quite well). Responses used a five-point Likert scale (0 = *not like me at all* to 4 = *very much like me*).

#### 2.2.2. Reactive and Proactive Aggression

Drawing on the work conducted by Little [37], we developed eight items aiming to measure adolescent aggression. This scale assesses both reactive (5 items, e.g., When someone angers me, I treat them indifferently or stop talking to them; average mean extracted AVE = 0.51; McDonald’s Omega ω = 0.71), and proactive aggression (3 items, e.g., I threaten others to get what I want; AVE = 0.52; ω = 0.73). A Likert-type response format was used (0 = never to 4 = always). The CFA results confirmed a good fit of the measurement model (*X*^2^= 44.74, *df* = 19, *p* = 0.002; Bollen–Stine bootstrap *p* = 0.07; SMRM = 0.04, TLI = 0.93; CFI = 0.96; RMSEA = 0.05, 90% CI (0.04, 0.08)).

### 2.3. Procedure

First, the study received ethical clearance from the Ethical Research Committee from the Technological Institute of Sonora (Authorization number: PROFAPI_ 2020_0018). Then, we gained authorization from school authorities for conducting the study. In a virtual meeting organized by the teachers, we informed the students’ parents about the research purpose. Then a consent letter was sent by email to parents to request their authorization for their children to respond to the questionnaires. Only 3% of parents rejected their children’s participation. Once approvals were gained, students were invited to participate in the study voluntarily. Data collection was carried out through online surveys. The time estimated to respond to the survey was about 20 to 30 min.

### 2.4. Data Analysis

We verified that missing data (less than 5%) were completely random. We treated missing data using multiple imputation methods, accessible in SPSS 25 (IBM Corp., Armonk, NY, USA). Descriptive statistics were run on the items (means, standard deviations, skewness, and kurtosis). Then, an unconditional random effect model was calculated to examine the school dependency of temperance and bullying aggression. The results suggested that temperance differences (Wald *z* statistic = 1.68, *p* = 0.092; intraclass coefficient ICC = 0.04) and aggression (Wald *z* statistic = 1.24, *p* = 0.214; ICC = 0.05) differences were not dependent on school [61,62]. Confirmatory factorial analyses (CFA) were conducted using the Bollen–Stine and maximum likelihood bias-corrected confidence bootstrapping estimator (500 replicates with 95% CI) in AMOS 25 (IBM Corp., Armonk, NY, USA). These estimators were chosen as the Mardia coefficient value was 9.47, which suggests multivariate non-normality. Bootstrapping is a robust procedure for dealing with non-normality in multivariate data [63,64,65].

#### 2.4.1. Dimensionality

In order assess the dimensionality of the temperance scale, we analyzed a first-order factor goodness-of-fit model (Model A). After establishing the four first-order measurement model’s adjustment, we tested a model with these four factors as indicators of a second-order temperance dimension to assess whether this first-order model could be conformed with the dimensions of one second-order factor model (Model B). In estimating the models’ global goodness of fit, we used the X^2^ statistic and associate probability, and Bollen–Stine bootstrap probability. Since X^2^ and Bollen–Stine bootstrap are sensitive to large samples [66,67,68], the standardized root means square residual (SRMR), comparative fit index (CFI), Tucker–Lewis index (TLI), and root mean square error of approximation (RMSEA) with their confidence intervals were reported. Structural equation modeling (SEM) literature suggests that model fit is adequate when X^2^ with *p* > 0.001; Bollen–Stine *p* < 0.05; CFI ≥ 0.95, and TLI ≥ 0.90. For the SRMR and RMSEA, a value ≤ 0.05 shows that the model fit is excellent, and a value ≤ 0.08 indicates an acceptable fit [38,69]. Differences in X^2^ (ΔX^2^) and the Bayesian information criterion (ΔBIC) were utilized to compare models. In cases where resulting differences in the X^2^ (ΔX^2^) value are significant, the model with a lower X^2^ has a better fit to the data [38,70]. Differences of BIC > 10 show distinctions in the model’s fit to the data, and a model with greater BIC has a poorer fit [38,71,72].

#### 2.4.2. Reliability 

Reliability was tested using average variance extracted (AVE) and McDonald’s Omega (ω). Values of AVE ≥ 0.50 and ω ≥ 0.70 were assumed as the indicators of adequate reliability [73,74,75].

#### 2.4.3. Measurement Invariance

Nested models were tested according to the procedure suggested in the literature [76,77]. We tested the baseline model configural that considered a fixed number of factors in each group (configural invariance). When the baseline model fit each group, we tested the factors’ loading invariance across groups (metric invariance). Once the metric invariance was verified, we evaluated the invariance-constrained measurement intercept (scalar invariance). Differences in X^2^ with an associated *p* < 0.001 suggest the measurement model is equivalent across groups [38,77]. However, the ΔX^2^ statistic is sensitive to sample sizes [77,78]; thus, scholars have advocated using goodness-of-fit indexes, such as differences in CFI (ΔCFI) and differences in RMSEA (ΔRMSEA). We followed the values proposed by scholars [77,79], who assert that differences greater than 0.01 in the CFI and 0.015 in the RMSEA exhibit a significant difference in model fit for the testing of invariance. In cases where the two procedures differ, we relied on the values of differences in CFI and RMSEA because of the larger sample used in this study [77,78,79]. If scalar invariance was confirmed, we calculated groups’ latent mean differences. For this, the means for the reference group (male and early adolescents) were fixed. We used a z statistic to compare latent means [38,76].

#### 2.4.4. Discriminant Validity

Discriminant validity confirms that the constructs are empirically unique [80,81]. Campbell [82] suggests that it ensures that a latent variable is “not correlated too highly with measures from which it is supposed to differ” (p. 6). Based on the literature, we assumed that discriminant invariance is confirmed when the average variance extracted (AVE) in each factor is greater than the square of this correlation with the other scale factors [81,83].

#### 2.4.5. Concurrent Validity

Concurrent validity requires that the scale scores correlate in a hypothesized model with other constructs measured simultaneously [84]. To test concurrent validity, correlations to temperance dimensions with aggressive and proactive bullying aggression were calculated. Values of *r* greater than 0.10 indicate smaller effects, *r* values between 0.20 and 0.29 reveal a medium effect, and *r* values greater than 0.30 suggest a large effect [85].

#### 2.4.6. Model Cross-Validation

We used a cross-validation method to test the replicability of the model dimensionality obtained in the calibration sample (*n* = 430) in an independent sample of adolescents (*n* = 430). A multigroup analysis was used to assess the model replicability in an independent sample. We compared the unconstrained model with a model that had factor loadings and fixed variances/covariances. Based on the SEM literature, we considered that factorial invariance was confirmed when ΔX^2^ was not significant (*p* > 0.001), ΔCFI ≤ 0.01, and ΔRMSEA ≤ 0.05. The X^2^ statistic is sensitive to a larger sample and non-normality departures, so we used ΔCFI and ΔRMSEA values when results were contradictory.

## 3. Results

### 3.1. Descriptive Item Analysis

The collected responses suggested that adolescents exhibit a moderate level of temperance. Values’ skewness and kurtosis indicated normal univariate distribution in all items (see Table 2).

### 3.2. Dimensionality

The initial four-first-order factor model (Model A) did not fit the data (see Table 3). Therefore, we improved the model’s fit based on the analysis of factor loadings and modification indices. The literature suggests that the factor loading for an item should be 0.6 or higher [67,74,86] to be a salient factor. Based on this, item 1 (“I often stay mad at people even when they apologize”; standardized factor loading = 0.11), item 5 (“I am not a show-off”; standardized factor loading = 0.04), item 10 (“I often find myself doing things that I know I shouldn’t be doing”; standardized factor loading = 0.42), and item 14 (“My temper often gets the best of me”; standardized factor loading = 0.17) were removed from the model. In addition, considering the modification indices (MI > 5) and the theoretical issues [38,74], we added three error covariances.

These changes resulted in a significant improvement in the fit of this model (see Table 4). The goodness-of-fit suggests an acceptable fit of the four-first-order factors model (Model B). Then we compared the four-first-order factor models (Model B) with one second-order model (Model C) that displayed four first-order factors. The adjustment to the data of one second-order factor model (Model C) was statically better than that of the four first-order factor model, ΔX*^2^* = 11.25, *df* = 2, *p* < 0.001; ΔBIC = 11.25. Therefore, based on theoretical and empirical findings, which suggest that temperance is a virtue that comprises several strengths, we chose Model C over the other choices and the described results are based on this model.

**Figure 2:**
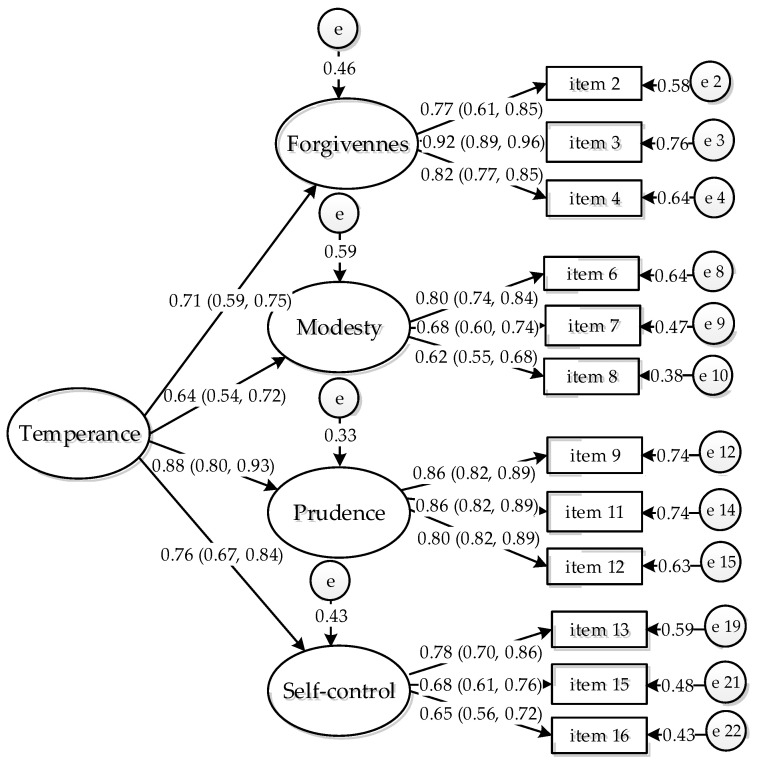
Final factor model of temperance depicting a one-dimensional second-order factor. Note: The 95% CI of the standardized factor loadings are represented in parentheses.

The estimated standardized factor and confidence interval (95%) for the one second-order measurement model are presented in Figure 2. The values of standardized factor loadings ranged from 0.54 to 0.92 and were statistically significant (*p* < 0.001). We found that the one second-factor model, X^2^ (*df* = 97) = 125.74, *p* = 0.019; Bollen–Stine *p* = 0.10; CFI = 0.98; TLI = 0.97; RMSEA = 0.029, 90% CI (0.015, 0.044) fit better to the data. Additionally, the expected bivariate correlations between the factors of forgiveness, modesty, prudence, and self-control were positively correlated with each other (*p <* 0.001). These correlations ranged from moderate to high (0.34 to 0.58). The reliability was acceptable: temperance (AVE = 0.60, ω = 0.82), forgiveness (AVE = 0.70, ω = 0.83), modesty (AVE = 0.50, ω = 0.72), prudence (AVE = 0.71, ω = 0.82), and self-control (AVE = 0.52, ω = 0.70).

### 3.3. Measurement Invariance by Gender

To assess the gender invariance of the second-order measurement model (Model C), a nested model was tested (see Table 4). The baseline model had a satisfactory fit for girls and boys (configural invariance), X^2^ = 228.95, *df* = 188, *p* = 0.022; Bollen–Stine bootstrapping *p* = 0.055; SRMR = 0.06; TLI = 0.98; CFI = 0.98; RMSEA = 0.024, 90% CI (0.010, 0.035). Then, we examined the constrained model with all factor loadings constrained (metric invariance). The differences in X^2^ statistics between models were not statistically significant, ΔX^2^ = 19.53, Δ*df* = 12, *p* = 0.076, and ΔCFI and ΔRMSEA were smaller than 0.01 and 0.015, respectively, ΔCFI = 0.001, ΔRMSEA = 0.002. Therefore, the data supported metric invariance by gender. In addition, we added constraints to the intercepts to be equal (scalar invariance), the ΔX^2^ = 53.75, Δ*df* = 41, *p* = 0.088, and changes in CFI and RMSEA were not significant (ΔCFI = 0.004, ΔRMSEA = 0.001), indicating no meaningful differences in the intercepts of the observed variables between groups.

### 3.4. Measurement Invariance by Stage of Adolescence

The baseline model fit to the data (configural invariance), X^2^ = 225.16, *df* = 186, *p* = 0.026; Bollen–Stine bootstrapping *p* = 0.052; SRMR = 0.05; TLI = 0.98; CFI = 0.98; RMSEA = 0.024, 90% CI (0.009, 0.034), supporting the equivalence of the second-order factor structure of temperance across early and middle adolescent groups. Then, we assessed the metric invariance of all factor loadings (measure invariance). The model with the factor loadings constrained fit adequately to the data based on the criteria of the X^2^ differences and changes in CFI and RMSEA values, ΔX^2^ = 11.82, *df* = 12, *p* = 0.46; ΔCFI = 0.001; ΔRMSEA = 0.001), which suggests that the factor loadings are consistent across the stages of adolescence. Finally, we constrained the intercepts (scalar invariance) in the model comparison. Our findings suggested that there are no important group differences in the intercept, ΔX^2^ = 44.52, *df* = 41, *p* = 0.327; ΔCFI = 0.002; ΔRMSEA = 0.002. The goodness-of-fit statistic suggested that the measurement model was invariant across early and middle adolescent groups (see Table 4).

### 3.5. Latent Means Differences

To test latent means differences, we fit males’ means to zero. The analysis revealed significant mean differences by gender on three of the first-order factors. Females had higher scores on forgiveness and modesty than males, but lower scores on self-regulation than males. The gender difference in prudence was not statistically significant.

Regarding latent means differences by adolescence stage, we chose early adolescents as the reference group and estimated the latent mean of the middle adolescent group. The test revealed that differences in forgiveness, prudence, and self-control were not statistically significant. However, the mean difference in modesty was statistically significant (see Table 5). Middle adolescents had a higher score on modesty than early adolescents.

### 3.6. Concurrent Validity

The dimensions of temperance correlated as expected with proactive and reactive aggression (see Table 6). As anticipated, all the factors of temperance had a negative correlation to proactive and reactive bullying aggression. The effect size of the correlation between modesty and proactive and reactive aggression was small (*r* > 0.10), and the values of all other correlations indicated a medium (*r* > 0.20) or large (*r* > 0.30) effect size. Overall, these results suggest that correlations between temperance dimensions and both types of aggression have theoretical and practical implications [83], confirming the Temperance Scale’s concurrent validity.

### 3.7. Cross-Validation Analysis

We cross-validated the data to address problems associated with the replicability of the model. The model was tested on an independent sample. Multigroup invariance analysis provided evidence of configural (X^2^ = 60.21, *df* = 48, *p* = 0.111; SRMR = 0.06; CFI = 0.96; TLI = 0.95; RMSEA = 0.05, 90% CI [0.03, 0.07]), metric, and scalar invariance (see Table 7). This evidence allowed us to conclude that the measurement model is replicable in both samples.

## 4. Discussion

We analyzed the psychometric properties of one second-order multidimensional model of Temperance of VIA-Youth, according to Park and Peterson’s [20] conceptualization. Given the gaps in the construct measurement, this study can add to the field, particularly in terms of temperance assessment. Overall, our results showed that the adjustment to a single second-order measurement model fit the data better and demonstrated its replicability through cross-validation. Moreover, the results supported measurement invariance, indicating that the measurement model is equivalent by gender and adolescence stage. For deeply understanding the underpinning differences around temperance, this characteristic of the scale is crucial. Finally, we confirmed the discriminant and concurrent scale validity.

### 4.1. Temperance as a Second-Order Factor

The results confirmed our second-order structure hypothesis, which comprises four first-order factors: forgiveness, prudence, modesty, and self-regulation. Furthermore, after comparing the first-order and second-order models, we found evidence suggesting that the second-order model fits better to the data. These findings are aligned with previous research [21,23], indicating that temperance has a second-order structure that emerges from its four strengths. Considering this, subsequent investigations should analyze the foundations and outcomes of temperance considering its four dimensions.

### 4.2. Measurement Invariance by Gender and Adolescence Stage 

Our findings support the measurement equivalence of the Temperance Scale by gender and stage of adolescence. These results indicate that the scale items may be utilized to measure this construct in both genders and in early vs. middle adolescents. Therefore, unlike previous scales, this scale allows researchers to compare genders and stages of adolescence more fairly and meaningfully.

Latent mean differences indicate that females scored higher in forgiveness and modesty than males. These results are in alignment with previous research [29,33]. Furthermore, similarly to other studies [31,32], we found that males showed higher self-control than females. Data did not show differences in forgiveness, prudence, and self-control regarding the adolescence stage. These findings are also congruent with past studies [32,35] that have found no relation between temperance and age. However, our findings reveal that middle adolescents scored higher in modesty than early adolescents. This evidence is consistent with that of Brown et al. [32], who found higher levels of modesty in older adolescents. Regardless of the present results, further studies should continue exploring gender and age differences to clarify the underpinnings of these discrepancies and their implications on adolescent development.

### 4.3. Discriminant Validity

The results prove that each temperance subscale assesses a different scale dimension, which supports discriminant validity. In line with previous research, study results indicate that temperance dimensions evaluate a different strength [20,21]. Our study provides empirical and theoretical evidence of the multidimensionality of temperance. Further studies need to examine the variables associated with first-order dimensions of temperance and its consequences in relation to adolescents’ psycho-emotional development on each dimension of temperance.

### 4.4. Concurrent Validity

In addition, the data provide evidence in favor of concurrent validity. In line with prior research [45,52,87], these results showed significant and negative associations between traits that conformed to temperance virtues and proactive and reactive aggression. Moreover, these correlation effect sizes suggest practical implications. Overall, these results indicate that temperance and its strengths may be important variables to consider for preventing peer aggression.

### 4.5. Theoretical and Practical Implications 

The results of this study suggest that theory about virtues and character strengths is a generative framework to study positive behavior. Furthermore, our findings confirm that virtues conform to strengths that influence moral behavior [2,20]. Specifically, the study evinced that temperance is a second-order factor that displays first-order factor measures: forgiveness, modesty, prudence, and self-regulation. Similarly to other studies [21,23], these findings confirmed this factor structure. The study confirms the value of the original classification of character strengths in the VIA. This instrument will allow us to analyze the possible positive results of temperance and explore the threshold effects and the possible exponential effects of combining two or more strengths [88]. In addition, our findings suggest that strengths that conformed to temperance virtues are essential for reducing peer aggression and should contribute to the comprehension of the underpinning factors of bullying. In this regard, temperance strengths are crucial for protecting people from excesses and encouraging positive social relations and adaptive behaviors [2], which could help to decrease peer aggression.

From a practical perspective, the present study highlights the value of a scale with robust psychometric properties to measure temperance in adolescents. The accurate measurement of temperance is critical for practitioners and schools in order to enhance adolescents’ strengths rather than their weaknesses, thereby improving their mental health and fostering positive development [88]. Furthermore, latent means differences could support the development of differentiated tools to increase these strengths at different stages of adolescence and by gender, offering the opportunity to direct more appropriate strategies to encourage adolescents to engage in this virtue. Overall, robust theoretical and psychometrically temperance measures allow researchers to generate relevant findings regarding the antecedents and consequences of temperance in adolescence.

### 4.6. Limitations

Although this study provides a helpful scale for researchers, some limitations must be considered. First, data collection was carried out through self-reports; therefore, the students’ responses could be influenced by social desirability [89]. Second, our sample consisted of adolescents from northwestern Mexico; therefore, a more diverse sample is desirable to generalize the results, recognizing that student responses may differ according to the country or region. Third, cross-cultural studies are essential to assess the replicability of the measurement model in a culturally diverse population. Forth, longitudinal designs are necessary to assess the extent to which temperance changes in childhood and adolescence across time and in terms of its relationships with bullying aggression.

## 5. Conclusions

The present research sheds light on the current understanding of temperance as a virtue and the strengths that comprise it. Our findings confirmed the value of the theoretical scheme of temperance [20] as a multifactorial second-order construct. Given the importance of having appropriate measures for evaluating constructs in positive psychology, this scale provides a robust psychometric instrument for assessing temperance in adolescents. We believe that this virtue is crucial for the positive development of youth. Therefore, we consider that future studies should explain the means through which temperance is built in school and family environments.

Additionally, our study provides a valuable instrument with evidence of robust validity for the evaluation of temperance as a multidimensional construct. The above allows better understanding to assess each of these strengths in particular, as well as helping to promote them in school interventions with adolescents.

## Figures and Tables

**Table 1:** Temperance scale items.

Item	Forgiveness	Modesty	Prudence	Self-Control
1. I often stay mad at people even when they apologize. *	✓			
2. I forgive people if they say they are sorry for hurting me.	✓			
3. I am a forgiving person.	✓			
4. When someone apologizes, I give them a second chance.	✓			
5. I am not a show-off. *		✓		
6. I don’t boast about what I achieve.		✓		
7. I let other kids talk about themselves rather than focusing the attention on me.		✓		
8. I don’t come across like I am better than others.		✓		
9. I review the consequences of my behavior before I take action.			✓	
10. I often find myself doing things that I know I shouldn’t be doing. *			✓	
11. I think carefully before I act.			✓	
12. I am cautious not to do something that I will regret later.			✓	
13. I have a lot of patience.				✓
14. My temper often gets the best of me. *				✓
15. When I really want to do something right now, I am able to wait.				✓
16. I am able to control my anger really well.				✓

Note: ✓—final items; *—removed items.

**Table 2:** Temperance scale items’ descriptive statistics.

Item	*M*	*SD*	Skewness	Kurtosis
Item 1	1.64	1.25	0.32 (0.12)	−0.84 (0.25)
Item 2	2.78	1.15	−0.83 (0.12)	−0.06 (0.25)
Item 3	2.80	1.16	−0.81 (0.11)	−0.69 (0.25)
Item 4	2.71	1.17	−0.71 (0.12)	−0.07 (0.25)
Item 5	1.85	1.19	0.08 (0.12)	−0.84 (0.25)
Item 6	1.88	1.21	0.03 (0.12)	−0.75 (0.25)
Item 7	2.28	1.31	−0.29 (0.12)	−0.93 (0.25)
Item 8	2.11	1.39	−0.14 (0.12)	−1.21 (0.25)
Item 9	2.43	1.25	−0.39 (0.12)	−0.76 (0.25)
Item 10	1.64	1.19	0.25 (0.12)	−0.76 (0.25)
Item 11	2.41	1.21	−0.32 (0.12)	−0.71 (0.25)
Item 12	2.49	1.18	−0.41 (0.12)	−0.62 (0.25)
Item 13	1.90	1.29	0.13 (0.12)	−1.03 (0.25)
Item 14	2.08	1.24	-0.08 (0.12)	−0.85 (0.25)
Item 15	2.17	1.11	−0.28 (0.12)	−0.41 (0.25)
Item 16	2.01	1.23	−0.08 (0.12)	−0.88 (0.25)

**Table 3:** Goodness-of-fit statistics of the hypothesized first-order and second-order models.

Factor Model	Χ^2^	*df*	*p*	Bollen–Stine Bootstrap *p*	SRMR	CFI	TLI	RMSEA	BIC
A. Four first-order	428.16	116	< 0.001	0.005	0.11	0.91	0.89	0.086	700.55
B. Four first-order (adjustment)	136.99	95	0.003	0.06	0.06	0.98	0.98	0.031	379.78
C.Second-order	125.74	97	0.019	0.10	0.06	0.98	0.97	0.029	368.52

Note: Χ^2^—chi-square; *df*—degrees of freedom; *p*—associated probability; SRMR—standardized root mean square residual; CFI—Comparative fit index; TLI—Tucker–Lewis index; RMSEA—Root mean square error of approximation; BIC—Bayesian Information Criterion.

**Table 4:** Summary of measurement invariance results of one second-order dimensional model of temperance scale (*n* = 430).

Model	Χ^2^	*df*	Δχ^2^	Δ*df*	*p*	ΔCFI	ΔRMSEA
Gender							
Configural	228.95	188					
Metric	248.48	202	19.53	12	0.076	0.003	0.002
Scalar	282.70	229	53.75	41	0.088	0.004	0.001
Stage of Adolescence (early vs. middle)							
Configural	225.11	186					
Metric	236.94	198	11.82	12	0.460	0.001	0.001
Scalar	269.61	227	44.50	41	0.327	0.002	0.002

Note: *df*—degree free; Δχ^2^—difference in chi-square; Δ*df*—difference in degree free; ΔCFI—difference in comparative fit index; ΔRMSEA—difference in root mean square error of approximation.

**Table 5:** Latent means differences by gender and adolescence stage.

Variable Factor		*M*	*z*-Statistics	*p*	Cohen’s *d*
	Forgiveness	0.23	3.29	0.015	0.23
Gender	Modesty	0.32	3.84	0.004	0.27
	Prudence	0.04	−0.42	0.687	0.01
	Self-control	−0.23	−3.14	0.019	0.22
	Forgiveness	−0.01	−0.21	0.908	0.01
Adolescence stage	Modesty	0.31	3.67	0.007	0.25
	Prudence	0.09	1.26	0.357	0.08
	Self-control	0.03	0.41	0.753	0.03

**Table 6:** Means, standard deviations, and correlations of dimensions of the temperance scale with measures of aggression.

			Measure	
Dimension	*M*	*SD*	Proactive Aggression	Reactive Aggression
Temperance	2.34	0.76	−0.24 ***	−0.24 ***
Forgiveness	2.76	1.03	−0.21 ***	−0.24 ***
Modesty	2.06	1.12	−0.13 **	−0.11 *
Prudence	2.45	1.01	−0.24 ***	−0.21 ***
Self-control	2.08	0.93	−0.28 ***	−0.34 ***

* *p* < 0.05. ** *p* < 0.01; *** *p* < 0.001.

**Table 7:** Results of comparison across calibration (*n* = 430) and validation sample (*n* = 430)**.**

Model	X^2^	*df*	ΔX^2^	Δ*df*	*p*	ΔCFI	ΔRMSEA
Configural	60.21	48			0.111		
Metric	65.58	55	5.37	7	0.567	0.001	0.003
Scalar	72.91	65	7.33	10	0.694	0.001	0.002

## Data Availability

The data presented in this study are available on request from the corresponding author.
